# Carvacrol partially reverses symptoms of diabetes in STZ-induced diabetic rats

**DOI:** 10.1007/s10616-013-9563-5

**Published:** 2013-04-12

**Authors:** Gokhan Bayramoglu, Hakan Senturk, Aysegul Bayramoglu, Mustafa Uyanoglu, Suat Colak, Ayse Ozmen, Durdane Kolankaya

**Affiliations:** 1 Science and Arts Faculty, Department of Biology, Artvin Coruh University, 08000 Artvin, Turkey; 2 Science and Arts Faculty, Department of Biology, Eskisehir Osmangazi University, 26480 Eskisehir, Turkey; 3 Science and Arts Faculty, Department of Biology, Hitit University, 19030 Corum, Turkey; 4 Science Faculty, Department of Biology, Hacettepe University, 06800 Ankara, Turkey

**Keywords:** Carvacrol, Diabetes, Streptozotocin, Rat, Liver

## Abstract

Little is known about the protective effects of carvacrol on the symptoms of streptozotocin induced diabetes in rats. Hence, this present study was designed to evaluate the protective effect of the strong antioxidant, carvacrol, on the symptoms of streptozotocin induced diabetes in rats. Carvacrol at the doses of 25 and 50 mg/kg body weight were orally administered to diabetic rats for a period of 7 days after the onset of diabetes. Food-water intake and body weight changes were daily recorded. Biochemical parameters such as serum glucose, insulin, total cholesterol, alanine aminotransferase, aspartate aminotransferase and lactate dehydrogenase were measured. Although treatment of diabetic rats with oral administration of carvacrol resulted in a slight reduction in serum glucose level and significant reduction in serum total cholesterol, alanine aminotransferase, aspartate aminotransferase and lactate dehydrogenase in comparison with diabetic control rats, there were no significant differences in serum insulin levels, food-water intake values and body weight changes. Despite the inadequacy of carvacrol on diabetes treatments, it was determined to have at least a partially protective role on liver enzymes.

## Introduction

Diabetes is a severe disease caused by autoimmune insulin deficiency (type 1) or insulin resistance (type 2). The concomitant hyperglycemia and/or hypoinsulinemia have serious detrimental effects on the body’s condition (Hoybergs et al. 2008). Also diabetes is associated with disturbances in carbohydrate, protein and fat metabolism which occur secondary to an absolute or relative lack of insulin (hypoinsulinemia) (Schmatz et al. 2012). In addition, several authors have reported increases in alanine aminotransferase (ALT), aspartate aminotransferase (AST) and lactate dehydrogenase (LDH) activities as well as changes in lipid concentration in the diabetic patients’ serum (Juśkiewicz et al. 2008; Sepodes et al. 2004; Bi et al. 2008).

The goals of managing diabetes mellitus are to optimize the control of blood glucose level, reduce the oxidative stress effects and normalize disturbances in lipid metabolism (Saravanan and Ponmurugan 2012). Synthetic anti-diabetic agents can produce serious side effects, including hypoglycemic coma and disturbances of the liver and kidneys. Therefore, the search for more effective and safer antidiabetic agents continues to be an important area for research (Ali and Agha 2009).

**Fig. 1:**
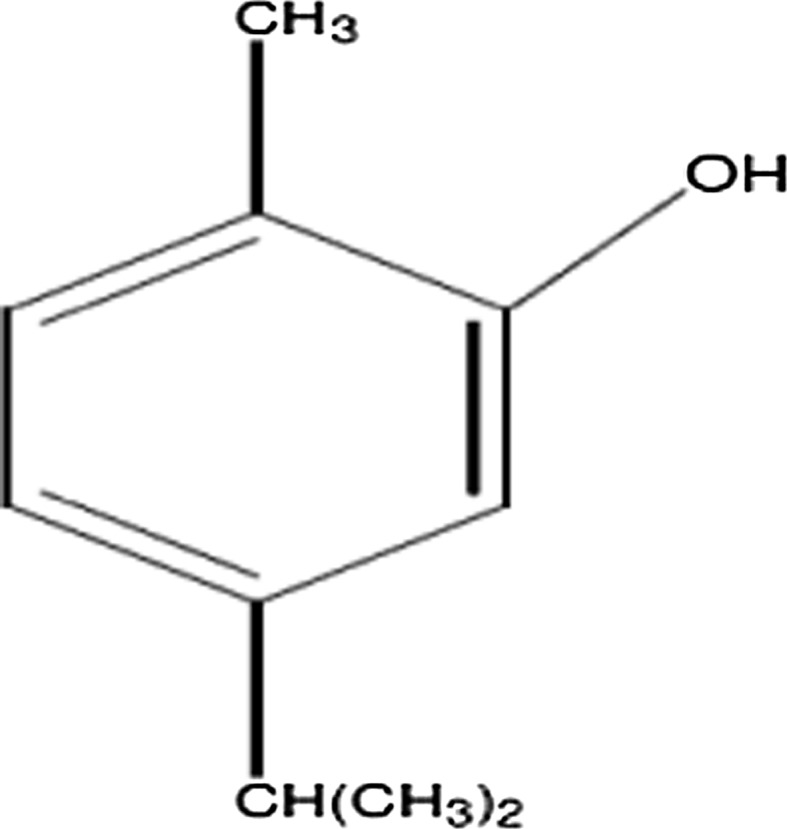
Chemical formula of carvacrol

For centuries, plants were used in folk medicine treatments due to their medicinal and protective abilities. Recent epidemiological studies show that consumption of fruits, vegetables, grains and legumes prevents chronic illnesses (Craig 1997; Miller et al. 2000; O’Keefe and Cordain 2004). This led to an increase in search of herbal products with anti-diabetic activity possessing fewer side effects (Habibuddin et al. 2008). Oregano water, a hydrosol of oregano, is used as a folk medicine in Turkey for liver health. Oregano oil contains carvacrol (CRV) as the main constituent (Canbek et al. 2008). CRV is a predominant constituent of essential oils, and is of the *Origanum* species. It is a monoterpenic phenol which has C_10_H_14_O closed chemical formula (Fig. 1). Synonyms are isopropyl-*o*-cresol, *p*-cymen-2-ol, 2-hydroxy-*p*-cymene, 5-isopropyl-2-methylphenol, iso-thymol (Uyanoglu et al. 2011).

To our knowledge, there is a very little information regarding the biological activity of CRV as an antioxidant agent against diabetes. Hence, the aim of the present study was to investigate the protective role of CRV at the doses of 25 and 50 mg/kg bw against type 1 diabetes in rats. For this purpose, body weight changes, daily food-water intake values and serum glucose, insulin, total cholesterol (TC), ALT, AST and LDH levels were measured and compared statistically.

## Materials and methods

### Plant extract

The plant substance tested in this study, CRV, was isolated from steam distillated essential oil of *Origanum onites* L. collected from West Anatolia and these samples were kindly provided by the Medical Plants, Drug and Scientific Research Center, Anadolu University, Eskisehir, Turkey (AUBIBAM). For the isolation, the fractional distillation method was performed by using a lab-size glass fractional distillation unit containing a column packed with S/S Knit Mesh packing material (2.8 cm × 1.35 m). The reflux ratio was adjusted as 10/1–20/1 and the medium pressure was 8–10 mmHg. CRV-rich fractions were bulked to obtain CRV with 99 % purity (GCMS) (Canbek et al. 2008; Uyanoglu et al. 2011).

### Animals

Thirty-two adult *Spraque*-*Dawley* race rats (weighing 195–215 g) were obtained from TICAM (Medical and Surgical Experimental Research Centre, Eskisehir Osmangazi University) and housed in polycarbonate cages in an air-conditioned room (22 ± 2 °C) with a 12 h light/dark cycle (0700 hours on, 1900 hours off). Standard rat feed and water were provided ad libitum. They were allowed to acclimatize to the laboratory environment for 7 days before the start of the experiment.

All procedures were conducted in conformity with the Institutional Ethical Committee for Animal Care and Use at Eskisehir Osmangazi University (protocol number: 37/215) and the international guidelines on ethical use of animals (NIH publications No: 80–23).

### Experimental induction of diabetes

A freshly prepared solution of STZ (50 mg/kg bw) (purchased from Sigma, St Louis, MO, USA) in 0.1 M citrate buffer (pH 4.5) was injected intraperitoneally in a volume of 1 ml/kg. STZ-induced animals exhibited massive glycosuria and hyperglycemia within 2 days. DM was confirmed in STZ rats by measuring the fasting blood glucose concentration 96 h after the injection of STZ. The rats with blood “glucose level >200 mg/dl” were considered to be diabetic and were used in the experiment (Vijayakumar et al. 2006).

### Experimental design

The rats were randomly divided into 4 groups as follows (n = 8 per group):

- **Group 1 (NC):** Non-diabetic control rats,
- **Group 2 (DC):** Diabetic control rats received vehicle solution (olive oil, volume: 1 ml/kg bw),
- **Group 3 (CRV-25) and Group 4 (CRV-50):** Diabetic rats treated with CRV at the doses of 25 and 50 mg/kg bw (in vehicle solution), respectively

The vehicle (olive oil, volume: 1 ml/kg bw) and the CRV solutions (dissolved in olive oil, volume: 1 ml/kg bw) were administered orally using an intragastric tube daily for 7 days. Food-water intake and body weight changes were recorded daily during experimental period. After the treatment, the rats were fasted overnight and then under ether anesthesia, the blood samples from rats were intracardially collected in polystyrene tubes without anticoagulant. At the end of this application, rats were immediately sacrificed by cervical decapitation.

### Biochemical analysis

The serum samples were separated by centrifugation at 1,600*g* at 4 °C for 15 min using a cooling centrifuge (Hermle ZK510, Gosheim, Germany) and analyzed for the serum glucose, insulin, TC, ALT, AST and LDH.

The serum glucose, TC, ALT, AST and LDH levels were immediately measured with a commercial kit (Biolabo, Mons-en-Barœul, France) using an auto analyzer (Crony Instruments, Airone 200-RA, Rome, Italy). The serum glucose, TC, ALT, AST and LDH levels were expressed in “mg/dl, mg/dl, U/l, U/l and U/l”, respectively.

### Insulin assay

The serum insulin level of each blood sample was measured by an enzyme-linked immunosorbent assay using a commercial kit (ultrasensitive rat insulin enzyme-linked immunosorbent assay; Mercodia, Uppsala, Sweden) based on the direct sandwich technique in which two monoclonal antibodies are directed against separate antigenic determinants on the insulin molecule. The serum insulin levels were expressed in “μg/l”.

### Statistical analyses

All data are given as mean ± SD (standard deviation). Statistical analysis was performed with one-way ANOVA followed by Tukey post hoc test for multiple comparisons. Values of *p* < 0.05 were considered significant.

## Results

### Body weight (initial and final) and daily food-water intake values in groups

Table 1 depicts the values of body weight (initial and final) and food-water intake of all groups during the experimental period. There was a slight decrease between initial and final body weight in the groups of DC, CRV-25 and CRV-50. But this decrease was not statistically significant (*p* > 0.05). The food-water intakes in DC, CRV-25 and CRV-50 groups were significantly higher than those in NC group. However, there was no difference between DC and CRV-25 or CRV-50 (*p* > 0.05).

**Table 1:** Body weight (initial and final), daily food and water intake values in experimental groups

Groups^a^	Initial body weight (g)	Final body weight (g)	Daily food intake (g/100 g body weight)	Daily water intake (ml/100 g body weight)
NC	201.50 ± 3.81	205.75 ± 1.98	7.31 ± 0.32	16.49 ± 1.49
DC	206.50 ± 4.37	201.25 ± 3.19	11.77 ± 1.20^b^	52.03 ± 3.15^b^
CRV-25	208.25 ± 3.28	204.00 ± 3.20	12.54 ± 2.39^b^	50.88 ± 2.90^b^
CRV-50	207.25 ± 3.69	202.00 ± 5.85	10.75 ± 2.61^b^	48.74 ± 4.28^b^

^a^For details see “Materials and methods” section

Data are mean ± SD values (n = 8). *p* < 0. 05, significantly different from ^b^NC group by Tukey’s multiple range tests

### Change of the serum glucose and insulin levels

The serum glucose and insulin levels in all groups are shown in Table 2. The serum glucose levels in the CRV-treated groups were slightly decreasing and respectively reduced down to 3.14 and 6.89 % compared to the DC group rats. The serum glucose levels of DC and CRV-treated groups significantly increased as compared to NC group (*p* < 0.05).

**Table 2:** The serum glucose, insulin, TC, ALT, AST and LDH values in groups

Groups^a^	Glucose (mg/dl)	Insulin (μg/l)	ALT (U/l)	AST (U/l)	LDH (U/l)	TC (mg/dl)
NC	95.69 ± 6.06	6.04 ± 0.32	31.66 ± 2.40	91.50 ± 5.59	111.28 ± 12.27	62.35 ± 2.99
DC	427.17 ± 25.54^b^	2.47 ± 0.59^b^	87.34 ± 6.41^b^	174.37 ± 14.58^b^	277.58 ± 61.00^b^	98.87 ± 6.70^b^
CRV-25	413.75 ± 18.74^b^	2.42 ± 0.48^b^	61.70 ± 10.08^b,c^	106.18 ± 46.56^c^	177.52 ± 36.18^b,c^	78.55 ± 4.59^b,c^
CRV-50	397.70 ± 30.99^b^	2.83 ± 0.38^b^	58.17 ± 11.37^b,c^	121.04 ± 27.69^c^	162.25 ± 10.02^b,c^	72.95 ± 3.69^b,c^

^a^For details see “Materials and methods” section

Data are mean ± SD values (n = 8). *p* < 0. 05, significantly different from ^b ^NC group and ^c ^DC group by Tukey’s multiple range tests

The serum insulin levels in the DC and CRV-treated groups were significantly lower than those in the NC group. Moreover, there was no difference between DC and CRV-treated groups. With regard to the serum glucose and correlation between the non-diabetic and diabetic control (DC) group, the present study is in line with our earlier studies on diabetes (Yamac et al. 2008, 2009, 2010).

### The serum ALT, AST and LDH activities in groups

The serum ALT, AST and LDH activities in all groups are shown in Table 2. The serum ALT, AST and LDH activities were significantly increased in the DC group when compared to the non-diabetic normal control (NC) group (*p* < 0.05). The administration of CRV to STZ-induced diabetic rats significantly decreased the activities of these enzymes. Although the serum ALT and LDH level did not return to the basal level of non-diabetic NC group, the serum AST level was able to return (*p* < 0.05).

### Total cholesterol levels in groups

The serum TC levels in all groups are shown in Table 2. There was a significant increase in the levels of TC in DC rats when compared to non-diabetic NC rats (*p* < 0.05). The administration of CRV to diabetic rats significantly decreased the level of cholesterol. However, the serum cholesterol level did not return to the basal level compared to non-diabetic NC rats (*p* < 0.05).

## Discussion

In research investigations, type 1 diabetes is usually induced by a STZ injection and the animals often display typical characteristics of diabetes, i.e. polyuria, polydipsia, increased water and food intake, dehydration and weight loss (Hoybergs et al. 2008; Wang et al. 2010). The weight loss was associated with a correction in abnormalities due to osmotic diuresis and glucose intolerance, resulting from inadequate insulin secretion or hyperlipidemia in diabetes mellitus. Prolonged osmotic diuresis may cause excessive urinary electrolyte loss. Disturbances in renal function are associated with several abnormalities, including proteinuria and progressive renal failure (Hahm et al. 2011).

The present study is in line with our earlier studies on diabetes with regard to body weight change and food-water intake correlation between the non-diabetic and DC group (Yamac et al. 2009, 2010). Generally, the body weights are reduced in STZ-induced diabetic rats and recovered when subjected to hypoglycemic treatment (Hwang et al. 2005). Unfortunately, in our study, there has been no improvement in the symptoms of diabetes (body weight change and food-water intake) as a result of CRV administration (Table 1).

The mechanism by which STZ brings about its diabetic state includes selective destruction of pancreatic beta cells thereby causing the cells to be less active, leading to poor sensitivity of insulin for glucose uptake by tissues (Elisa et al. 2009). Effective control of blood glucose levels is a key step in preventing or reversing diabetic complications (Yamac et al. 2008). According to our data, the increased levels of serum glucose in STZ-induced diabetic rats were slightly lowered by administration of CRV (Table 2).

Generally, the plasma AST and ALT levels increase as a result of metabolic changes in the liver, such as administration of toxin, cirrhosis of the liver, hepatitis, and liver cancer. Thus, they can be used as markers to assess the extent of liver damage (Hwang et al. 2005). Epidemiological studies show that diabetic patients are at higher risk of chronic liver disease and hepatocellular carcinoma. Diabetes and insulin resistance were also identified as important factors associated with an increased risk of advanced liver fibrosis in patients with normal ALT (Kim et al. 2009).

In diabetes, several authors have reported increases in AST and ALT activities as well as changes in lipid concentration in the diabetic patients’ serum (Juśkiewicz et al. 2008). Moreover, LDH (a marker of nonspecific cellular injury), AST (a nonspecific marker for hepatic injury) and especially ALT (a specific marker for hepatic parenchymal injury) are used in the evaluation of hepatic disorders (Juśkiewicz et al. 2008; Sepodes et al. 2004; Bi et al. 2008). An increase in these enzyme activities reflects active liver damage (Elisa et al. 2009) and an increase in the activities of plasma ALT, AST, and LDH indicates liver dysfunction and moreover liver was necrotized in STZ-induced diabetic rats (Ohaeri 2001). Therefore, an increase in the activities of AST, ALT and LDH in plasma might be mainly due to the leakage of these enzymes from the liver cytosol into the blood stream (Navarro et al. 1993). Injury to the hepatocytes alters their transport function and membrane permeability, leading to leakage of enzymes from the cells. Therefore, the marked release of AST and ALT from liver cytosol into circulation indicates severe damage to hepatic tissue membranes during diabetes (Harris 2005). Therefore, increased activities of AST and ALT in this study may be interpreted as a result of liver cell destruction or changes in the membrane permeability indicating severe hepatocellular damage by diabetes. The treatment of CRV at the doses of 25 and 50 mg/kg bw per day was able to protect against increase in the activity of these enzymes in diabetic rats, demonstrating protective effect of this monoterpenic phenol against hepatic damage induced by diabetic state and it could be used as a drug to bring about hepatoprotective effect (Table 2).

The liver and some other tissues participate in the uptake, oxidation and metabolic conversion of free fatty acids, synthesis of cholesterol and phospholipids and secretion of specific classes of plasma lipoprotein (Kumar and Murugesan 2008) and the liver is regarded as one of the central metabolic organs in the body, regulating and maintaining lipid homeostasis. It has been demonstrated that insulin deficiency in diabetes leads to a variety of derangements in metabolic and regulatory processes, which in turn leads to accumulation of lipids in hepatic tissue (Saravanan and Ponmurugan 2012). Also, literature data have showed that diabetes is usually associated with abnormal high levels of serum lipids (Schmatz et al. 2012) and it is associated with profound alterations in the plasma lipid and lipoprotein profile. Accumulation of lipids in diabetes is mediated through a variety of derangements in metabolic and regulatory processes, especially insulin deficiency, thereby rendering the diabetic patient more prone to hypercholesterolemia (Sireesha et al. 2011). In diabetes, hypercholesterolemia is associated with the consequences of hyperinsulinemia, insulin resistance and glucose intolerance (Jeong et al. 2010).

Hypercholesterolemia and hypertriglyceridemia in STZ-induced diabetic rats are well documented. Excess production of serum fatty acids by STZ-induced diabetics promotes the conversion of excess fatty acids into phospholipids and cholesterol in liver (Saravanan and Ponmurugan 2012). In addition, plasma TC and triglyceride levels are also strongly related to the degree of DC rats. The increased TC and triglyceride levels observed in diabetic rats may be the result of impaired liver function caused by the damage done by STZ, which acts either directly or indirectly by enhancing the plasma glucose level (Table 2) (Hwang et al. 2005).

Generally, according to the literature, the cause of improvement in cholesterol levels in the blood depends on recovery of liver function. Furthermore, the reason for the recovery of liver function is related to presenting normal levels of blood glucose and insulin levels in the blood after treatment of diabetes. This relationship may not be enough to explain our findings clearly. Since the treatment of CRV in our study stimulated a slight increase in insulin levels and a decrease in glucose levels. According to our hypothesis, the improvement especially in the level of cholesterol may depend on the direct protection of CRV on the liver without presenting normal levels of blood glucose and insulin levels in the blood.

In conclusions, carvacrol used in STZ-induced type 1 diabetes model was unable to show the expected impact on pancreas. We believe that an increase in drug dose and treatment duration may possibly result in positive effect of carvacrol on pancreas. Although carvacrol did not show the expected impact on pancreas, no detrimental effect was observed. In this context, there is no harm to include carvacrol in the diet list of people with diabetes. Moreover, if we examine the topic in terms of positive effects of carvacrol on the liver, we believe that it will especially be useful for the diet of diabetics. In accordance with the parameters obtained in the present study, the use of carvacrol is beneficial for suppressing or delaying negative effects of STZ on the liver.
